# COVID-19 in Africa: care and protection for frontline healthcare workers

**DOI:** 10.1186/s12992-020-00574-3

**Published:** 2020-05-15

**Authors:** Matthew F. Chersich, Glenda Gray, Lee Fairlie, Quentin Eichbaum, Susannah Mayhew, Brian Allwood, Rene English, Fiona Scorgie, Stanley Luchters, Greg Simpson, Marjan Mosalman Haghighi, Minh Duc Pham, Helen Rees

**Affiliations:** 1 Wits Reproductive Health and HIV Institute, Faculty of Health Sciences, University of the Witwatersrand, Johannesburg, South Africa; 2 South African Medical Research Council, Cape Town, South Africa; 3 Department of Pathology, Microbiology and Immunology, Vanderbilt University Medical Center, Nashville, TN USA; 4 Division of Medical Education and Administration, Vanderbilt University School of Medicine, Nashville, TN USA; 5 Department of Global Health and Development, Faculty of Public Health and Policy, London School of Hygiene & Tropical Medicine, London, WC1H 9SH UK; 6 Division of Pulmonology, Department of Medicine, Stellenbosch University and Tygerberg Hospital, Cape Town, South Africa; 7 Division of Health Systems and Public Health, Department of Global Health, Faculty of Medicine and Health Sciences, Stellenbosch University, Stellenbosch, South Africa; 8 Department of Population Health, Aga Khan University, Nairobi, Kenya; 9 Department of Public Health and Primary Care, International Centre for Reproductive Health (ICRH), Ghent University, Ghent, Belgium; 10 Department of Epidemiology and Preventive Medicine, Monash University, Melbourne, Australia; 11 Burnet Institute, Melbourne, Australia; 12 Wildlife Forensic Academy, Buffelsfontein Nature Reserve, Cape Town, South Africa; 13 The University of Sydney, Faculty of Medicine and Health, Sydney, South Africa; 14 Disease Elimination, Burnet Institute, Melbourne, Australia; 15 Department of Epidemiology and Preventive Medicine, Monash University, Melbourne, Australia

**Keywords:** COVID-19, SARS-Cov-2, Africa, Human resources for health, Healthcare workers, Infection control, mental health

## Abstract

Medical staff caring for COVID-19 patients face mental stress, physical exhaustion, separation from families, stigma, and the pain of losing patients and colleagues. Many of them have acquired SARS-CoV-2 and some have died. In Africa, where the pandemic is escalating, there are major gaps in response capacity, especially in human resources and protective equipment. We examine these challenges and propose interventions to protect healthcare workers on the continent, drawing on articles identified on Medline (Pubmed) in a search on 24 March 2020. Global jostling means that supplies of personal protective equipment are limited in Africa. Even low-cost interventions such as facemasks for patients with a cough and water supplies for handwashing may be challenging, as is ‘physical distancing’ in overcrowded primary health care clinics. Without adequate protection, COVID-19 mortality may be high among healthcare workers and their family in Africa given limited critical care beds and difficulties in transporting ill healthcare workers from rural to urban care centres. Much can be done to protect healthcare workers, however. The continent has learnt invaluable lessons from Ebola and HIV control. HIV counselors and community healthcare workers are key resources, and could promote social distancing and related interventions, dispel myths, support healthcare workers, perform symptom screening and trace contacts. Staff motivation and retention may be enhanced through carefully managed risk ‘allowances’ or compensation. International support with personnel and protective equipment, especially from China, could turn the pandemic’s trajectory in Africa around. Telemedicine holds promise as it rationalises human resources and reduces patient contact and thus infection risks. Importantly, healthcare workers, using their authoritative voice, can promote effective COVID-19 policies and prioritization of their safety. Prioritizing healthcare workers for SARS-CoV-2 testing, hospital beds and targeted research, as well as ensuring that public figures and the population acknowledge the commitment of healthcare workers may help to maintain morale. Clearly there are multiple ways that international support and national commitment could help safeguard healthcare workers in Africa, essential for limiting the pandemic’s potentially devastating heath, socio-economic and security impacts on the continent.

## Methods for review of literature

### Inclusion and exclusion criteria

Studies were included if they presented data or commentaries on the infection risks and mental wellbeing impacts that healthcare workers face during the COVID-19 pandemic. Studies could be in any setting. We also included articles that covered the COVID-19 pandemic in Africa Iin general (these papers did not have to include infection risks and mental health). We excluded articles on infection control or mental health of COVID-19 if they covered topics that were not relevant to Africa.

### Search strategy

The literature was identified in a search on Medline(Pubmed) up to 24 March and identified using the following search strategy: ((((((coronavirus) AND ("2020"[Date - Create] : "3000"[Date - Create]))) OR SARS-CoV-2) OR 2019-nCoV) OR COVID).

## Background

Sustaining safe and quality care in the SARS-CoV-2 pandemic hinges on the health and mental wellbeing of frontline healthcare workers. Medical staff face exhaustion, difficult triage decisions, separation from families, stigma and the pain of losing patients and colleagues, in addition to their own risks of infection. In Italy as of 3 April 2020, around 10,000 healthcare workers have been infected and 74 have died, and many others have died in countries across the globe [1, 2]. While the pandemic in Africa is several weeks behind Europe and Asia, the number of cases in Africa is escalating fast [3–5]. Incidence varies considerably between countries in Africa possibly reflecting variations in volumes of air travel and differences in coverage of SARS-CoV-2 testing [6]. While many countries in Africa are stepping up their preparedness for COVID-19 [6], assessments by WHO point to substantial limitations in response capacity [7]. In particular, there are major shortages of human resources, critical care beds and laboratory capacity. For example, in 2018 the numbers of nurses or midwives to 10,000 population was about 6.0 in Côte d’Ivoire and Mozambique, around 11 in the Democratic Republic of the Congo and Kenya [8]. Corresponding figures for the United Kingdom were 81.7 and 132.4 in Germany. Many countries in Africa have fewer than 30 critical care beds to cover the entire population [9]. Notwithstanding these gaps, COVID-19 control efforts can draw on valuable lessons learnt during the recent Ebola outbreaks and from HIV prevention and treatment successes on the continent. In a similar way, countries such as Singapore and South Korea had learnt valuable lessons from their experiences of the SARS and MERS outbreaks, which they then applied to control of SARS-CoV-2.

In this review we describe the infection risks and mental health challenges that healthcare workers face in the COVID-19 pandemic and propose interventions to counter these in Africa. We highlight lessons from previous disease-control efforts on the continent and draw on experiences with SARS-CoV-2 in other parts of the world. We searched Medline (Pubmed) on 24 March (see Additional File Fig. 1 for the search strategy and PRISMA flow chart) and located 1464 articles, of which 88 were on healthcare workers, and 32 considered relevant to this review. Articles on healthcare workers and Ebola or HIV were identified in targeted searches.

### Healthcare workers’ vulnerability to COVID-19: critical interventions to minimise risk

In all settings, patients who have unusual symptoms of COVID-19 or very mild general flu-like symptoms pose considerable risk to healthcare workers who may not have a high level of clinical suspicion in these patients and adopt adequate protective measures. Additionally, in the tropics, patients may present with febrile conditions related to vector-borne and other infections, but have SARS-CoV-2 coinfection. In Thailand, for example, a patient was admitted with dengue fever and also had SARS-CoV-2 and the healthcare worker responsible for care became infected [10].

Healthcare workers – and, indeed, patients admitted to a health facility for other reasons – are particularly vulnerable to infection from ‘super-spreading events’. In one instance in Wuhan, China a large cluster of infections occurred in healthcare workers and patients [11]. These infections were traced to a patient with abdominal symptoms who had been admitted in the surgical department [11]. Tracing and controlling super-spreading events within hospitals requires resources, which may not be available in many Africa settings.

Risks of SARS-CoV-2 infection may be higher among professionals who work in close physical proximity to patients, such as ophthalmologists and dentists [12–15]. Additionally, some procedures such as non-invasive ventilation, high-flow nasal cannula and bag-mask ventilation may generate higher aerosol volumes, but be key treatment modalities in settings without mechanical ventilators [16].

Risks of healthcare worker infection can be mitigated with adequate precautions within health facilities [17–20]. Primarily, this involves the use of personal protective equipment (PPE) including a gown, gloves, face mask, and a face shield or goggles. Careful donning and doffing of this equipment remains a key defense, but requires considerable training and supervision. Risks for infection may also be highest at the beginning of the outbreak when healthcare workers may not yet be familiar with PPE use. There are major PPE shortages in high-income countries and it is likely that limited supplies will be allocated to less resourced countries [21]. These scarce PPE resources need to be appropriately used and distributed equitably across the globe – yet hoarding, misuse, intense competition between and within countries, price gouging, and export blocks are threatening to become the norm [21, 22]. Without international support, any reserves of PPE in hospitals are likely to be rapidly depleted in African countries and new supplies will be very difficult to secure [23]. Importantly, digitalized or telemedicine services could potentially reduce patient contact and thus risks for infection, and allow for national or international experts to give advice from a distance and support to less-experienced doctors. While there may be considerable costs in setting up such systems, these may be outweighed by savings in PPE, staff resources and improved patient outcomes. These initiatives may, however, be challenged by infrastructural constraints such as an unstable power supply or limited internet connectivity, and a lack of interoperability between digital systems.

Risks of infection in healthcare workers appears tied to duration of shifts and hand hygiene, among other factors [24]. Water supplies for handwashing, however, may be limited or unavailable in some parts of Africa. Guidance is needed on the range of low-cost cleaning agents that can be used in lieu of commercially-manufactured sanitisers for cleaning and disinfection of work surfaces and items such as pens, stethoscopes and mobile phones [25].

A range of simple low-cost interventions can reduce the likelihood of infection transmission to healthcare workers in hospitals, including facemasks for patients who have respiratory symptoms, tissues for patients, promotion of cough etiquette and hand washing, and maintenance of at least two metres distance from others. ‘Social [physical] distancing’ between patients and administrative, cleaning staff and healthcare workers may, however, be especially challenging in busy overcrowded primary health care clinics on the continent.

Many governments in Africa will require support from international donors to protect frontline healthcare workers from the outset of the pandemic. The government of China has provided personnel and equipment to support COVID-19 services in many parts of the world [26]. Support from China, building on already well-established links with countries across the continent, could play a major role in shaping the trajectory of the pandemic in Africa. Using international staff to provide care, however, presents challenges. In the Ebola outbreak in West Africa, for example, misunderstandings and language barriers were common with international medical teams, while the needs of local lower-level responders were sometimes neglected. The outsiders were viewed with distrust or suspicion, as, for example ‘patients were seen to be taken away by hazmat-suited strangers to die in unknown locations’ [27].

Mortality rates among healthcare workers who become infected may be especially high in many parts of Africa given the limited number of critical care beds [28]. Moreover, the large geographical distances pose tremendous practical difficulties in transferring ill healthcare workers from rural areas to secondary- or tertiary-level facilities in urban centres. Additionally, many healthcare workers in Africa themselves fall into the category of ‘high-risk’ groups for COVID-19, given their high rates of certain non-communicable diseases, tuberculosis, and HIV, although evidence on the impacts of the latter on COVID-19 is not yet available [29–31]. Importantly, the benefits of continuing to isolate healthcare workers with SARS-CoV-2 infection or high-risk exposures after a predefined time assuming they do not have symptoms, needs to be weighed against the consequent service disruptions in settings with severe human resource constraints [32].

Overall, much more thought is needed about the optimum use of healthcare workers in the COVID-19 response in rural parts of the continent. During the Ebola outbreak, mobile testing units moved between villages, detecting clusters of infection, quarantining those areas and building temporary treatment centres where required. Such efforts helped to target human and other resources to where it was most needed, while allowing economic activities to continue in unaffected areas. Some combination of antigen, antibody testing and symptom screening may be needed to identify affected communities who are then prioritised for COVID-19 services. Testing and later vaccinating of healthcare workers was a key strategy in the Ebola epidemic, but testing constraints and lack of a vaccine hinders that approach with the current pandemic. Serological testing which demonstrates past infection may provide major relief for healthcare workers and potentially allow people who have immunity to return to work.

### Securing the mental wellbeing of healthcare workers

Caring for patients with COVID-19 disease causes considerable mental stress, resulting in high levels of anxiety and post-traumatic stress disorders, especially among nurses [33]. These conditions have a major impact on healthcare workers, but also undermine their decision-making ability and quality of interaction with patients [34]. It is worth bearing in mind that the strain experienced at work is compounded by the very same disruptions and uncertainties felt by members of the general population at this time [35].

Psychological support is key, perhaps drawing on the large numbers of HIV counsellors or retired nurses in many parts of the African continent. Formal structured interventions, however, may encounter obstacles, given the competing priorities of health staff [36]. Informal mechanisms might be more successful, where, for example, counsellors or retired nurses visit healthcare worker rest areas to listen to difficulties or to the stories recounted by staff about their work. Support among healthcare workers through social media, such as the Vula platform in South Africa, may also relieve stress and could be extended to other countries [36]. WhatsApp groups among healthcare workers could provide advice on clinical decision making, but also be used to circulate messaging on mental health support. Regular communication between healthcare workers in the Ebola response meant that new knowledge could be shared among providers, especially where there were gaps in evidence on the disease [27].

While healthcare workers may accept an increased risk of infection as part of their chosen profession, they may have considerable anxiety about spreading the virus to their children, families and friends, especially those who are elderly or have chronic medical conditions, and perhaps even their pets, despite the evidence for this being limited [37, 38]. Healthcare workers may wish to have alternative accommodation to avoid the risk of household transmission. Student residences or hotels that are currently empty could be re-purposed to serve as places where they can rest and temporarily isolate themselves from their family [36]. Healthcare workers may also prefer to have longer shifts for a week and stay at the hospital during that time, rather than having shorter shifts and returning home each night. Protective actions within the home may include separating living spaces and bathrooms where possible and having a routine when arriving at home after duty, such as taking off shoes, removing and washing clothing, and immediately showering [39]. Many of these precautions may not be possible, however, in overcrowded areas with limited infrastructure and water availability, for example.

In any biological disaster, fear and stigmatisation are heightened, and healthcare workers may be a target of the latter. Many healthcare workers in the recent Ebola and SARS epidemics experienced considerable stigmatization, loneliness and even loss of trust within their own communities [40, 41]. In Singapore during the SARS epidemic in the early 2000s, for example, one nurse in a lift was told that her presence in the lift was spreading the virus to others, and another was scolded by fellow passengers for making trains “dirty”. These factors are of critical importance and healthcare workers need to feel socially supported; this may affect their self-efficacy, sleep quality and anxiety levels [42]. During the SARS epidemic, over time, some healthcare workers became reluctant to continue working [43, 44]. Maintaining staff motivation may be especially challenging where levels of trust in the health system and in the government are low, as may be the case in areas of Africa where governments are perceived to be corrupt [44]. A ‘risk allowance’ may be one strategy for motivating and retaining healthcare workers, although there might be jealousy as other staff will continue to care for non-COVID-19 patients and hence miss out on these benefits. Risk allowances were used during the recent Ebola epidemic in Western Africa where the benefits were apparent, but also the jealousies of those not receiving these allowances [45]. It is also important to acknowledge that healthcare workers are powerful agents for change and need to be involved in decision making and in shaping the outbreak response. Healthcare workers in Africa hold an authoritative voice in local communities and society more broadly. They can help ensure that local governing structures, national governments and the international community adopt effective control measures and treatment strategies, and prioritize their safety [46].

Initiatives to increase the number of healthcare workers available to meet the burden of care have included moving staff from other disciplines to medical wards, fast-tracking medical students to join the workforce, cancelling healthcare worker leave and drawing on retired healthcare workers [21, 47]. Healthcare workers can also be relieved from administrative tasks, freeing up their time for patient care [48]. Mobilizing existing HIV counselors and community healthcare workers may be a key strategy in the COVID-19 response in Africa. These cadres, who have long served as ‘first responders’ in Africa could be key information sources to dispel myths in the community, but also perform symptom screening and contact tracing. Community health volunteers and health committee members also provide important supports and linkages to the formal health system in many settings. During the Ebola epidemic, for example, these groups helped build isolation structures, performed tasks such as screening and contact tracing, and assisted healthcare workers to better understand the needs of the community. For their part, community volunteers’ main concerns included inadequate communication with the health system and concerns regarding compensation for their work [49]. Policymakers will, however, have to weigh up the benefits of ‘task shifting’ these community-based cadres against the risks of placing them in situations of heightened risk of acquiring the virus, and with little or no protective equipment.

## Conclusion

Healthcare workers in Africa face considerable challenges during the pandemic. We present 10 priority interventions to safeguard frontline healthcare workers in Africa (Table 1). The degree to which we protect their health and mental wellbeing will shape the pandemic in Africa, and thus its long-run impacts on social stability, economic growth, and security [53]. While there are clearly a range of priorities for the COVID-19 response on the continent, we strongly urge WHO, national governments, the private sector, and the general populace to pay concerted attention to healthcare worker safety and mental wellbeing.

**Table 1:** 10 priority interventions for securing the health and mental wellbeing of frontline healthcare workers in the COVID-19 response in Africa

1. Secure supplies of PPE of good quality.	
2. Capacity development on PPE use and infection control more generally.	
3. Research examining protection and support interventions for healthcare workers in Africa, including feasible approaches to repeated SARS-CoV-2 testing.	
4. Prioritize healthcare workers for SARS-CoV-2 testing, beds in ICU and other medical wards, drug and vaccine trials, and therapeutics when these become available.	
5. Politicians and other public figures to visit healthcare workers, acknowledge their commitment and sacrifices, and address any negative perceptions towards providers [50].	
6. Provide food and daily living supplies for healthcare workers, which save them time, but also demonstrate society’s appreciation of their work [51].	
7. Incorporate a range of healthcare worker cadres into the response, especially community healthcare workers and HIV counsellors.	
8. Creative interventions to reduce risks of infection, such as ‘Eagle-Eye Observers’ who are dedicated full-time staff charged with observing and correcting infection control errors, [52] a task which lay people could potentially do as this is largely based on checklists.	
9. Finances to cover the costs of funerals for healthcare workers.	
10. ‘Risk allowances’ to compensate healthcare workers for the risks they take and to motivate staff to continue to work.	

## Supplementary information

**Additional file 1.**

## Data Availability

Not applicable as it is a review. Data sharing is not applicable to this article as no datasets were generated or analysed during the current study.

## Abbreviation

PPE: Personal Protective Equipment
