# Supplementary material for: COVID-19 in Africa: care and protection for frontline healthcare workers
Source: Global Health. 2020 May 15;16:46. doi: 10.1186/s12992-020-00574-3 (PMC7227172; doi:10.1186/s12992-020-00574-3)
Supplement: Supplementary file 1 — Additional file 1. [file 12992_2020_574_MOESM1_ESM.docx]

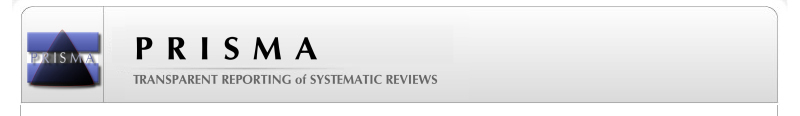
**PRISMA 2009 Flow Diagram**

Studies included in quantitative synthesis (meta-analysis)
(n=0)

Full-text articles excluded
(n=103)

Records excluded
(n=1320)

Records screened
(n=1456)

Records after duplicates removed
(n=1456)

## Identification

## Eligibility

## Included

## Screening

Records identified through database searching
(n=1464)

Additional records identified through other sources
(n=0)

Full-text articles assessed for eligibility
(n=136)

Studies included in qualitative synthesis
(n=33)
